# Diabetes Status Modifies the Association Between Cardiorespiratory Fitness and Coronary Heart Disease: A Cross‐Sectional Study

**DOI:** 10.1111/1753-0407.70176

**Published:** 2025-12-05

**Authors:** Li Wang, Yan Lu, Huifang Zhang

**Affiliations:** ^1^ First Clinical Medical College Shanxi Medical University Taiyuan Shanxi China; ^2^ Department of Cardiology First Hospital of Shanxi Medical University Taiyuan Shanxi China; ^3^ College of Public Health Shanxi Medical University Taiyuan Shanxi China

**Keywords:** cardiorespiratory fitness, coronary heart disease, cross‐sectional study, diabetes, peak oxygen uptake

## Abstract

- CRF is inversely associated with CHD risk in a dose–response manner.
- Diabetes status significantly modifies the association between CRF and CHD.
- High CRF levels were associated with a more pronounced reduction in CHD risk in diabetic participants than in non‐diabetic participants.

## Graphical abstract

In this cross‐sectional study, the protective effect of cardiorespiratory fitness (CRF) on coronary heart disease (CHD) risk differed between non‐diabetic and diabetic individuals, with a significantly greater reduction in CHD risk observed among participants with diabetes who had higher CRF levels. These findings highlight the importance of improving CRF for CHD prevention, particularly in diabetic individuals.

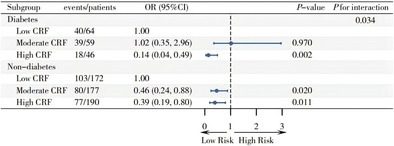

To the Editor,

Coronary heart disease (CHD) remains a leading contributor to the global disease burden [1]. Diabetes, an independent risk factor for CHD, increases the risk of CHD by 2–4 times [2]. Growing evidence indicates that cardiorespiratory fitness (CRF), designated as the “fifth vital sign” by the American Heart Association (AHA), is inversely associated with CHD risk [3, 4, 5]. However, individuals with diabetes often exhibit significantly reduced levels of CRF [6, 7]. Based on these findings, we hypothesize that the effect of CRF on CHD risk may differ by diabetes status. To test this hypothesis, we conducted a cross‐sectional study to examine whether the association between CRF and CHD is modified by diabetes status.

## 1 Methods

In this cross‐sectional study, we consecutively enrolled patients with suspected CHD admitted to the Department of Cardiology at the First Hospital of Shanxi Medical University from December 2021 to December 2023. Initial screening identified 797 eligible patients aged 18–75 years who had completed both cardiopulmonary exercise testing (CPET) and coronary angiography (CAG). After excluding 89 patients with severe heart failure (NYHA class III–IV), persistent ventricular arrhythmia, severe valvular heart disease, hypertrophic obstructive cardiomyopathy, significant congenital heart disease, pulmonary disease limiting exercise capacity, type I diabetes, anemia, autoimmune diseases, liver or kidney dysfunction, or malignant tumors, a total of 708 patients were included in the final analysis. This study was approved by the Medical Ethics Committee of the First Hospital of Shanxi Medical University (Approval Number: 2019‐K‐SK032) and complied with the Declaration of Helsinki. Informed consent was waived due to the anonymous nature of the data.

The exposure variable was CRF, expressed as peak oxygen uptake (VO_2_peak), assessed using gas analysis (CARDIOVIT CS‐200 ERGO; Schiller AG, Baar, Switzerland) during maximal symptom‐limited CPET on an electronically braked cycle ergometer [8]. The outcome variable was CHD, defined as ≥ 50% luminal stenosis in at least one major coronary artery based on CAG results [9]. Covariates are detailed in Table S1.

Confounders were selected based on their associations with the outcomes of interest or a change in effect estimate of more than 10% [10]. We used multivariate logistic regression models to investigate the association between CRF and CHD in the overall population. The results are presented as odds ratios (ORs) with 95% confidence intervals (95% CIs). To assess whether diabetes status modified the association between CRF and CHD, we conducted stratification analysis and interaction tests within the regression model. A two‐tailed *p*‐value < 0.05 was considered statistically significant. All statistical analyses were performed using EmpowerStats software (www.empowerstats.com; X&Y Solutions Inc. Boston, Massachusetts) and R software (http://www.R‐project.org; version 4.2.0).

## 2 Results

The mean age of the 708 participants was 56.97 ± 9.55 years, with 434 (61.30%) being men. CRF was categorized into three groups: low (first tertile), moderate (second tertile), and high (third tertile). Compared with the low CRF group, the risk of CHD was significantly reduced in both the moderate and high CRF groups across all models. The trend test showed a statistically significant result in all models (all *p* < 0.001), indicating a significant dose–response relationship between CRF and CHD (Table 1).

**TABLE 1:** Association between CRF and CHD.

CRF, mL/min/kg	Events/patients	Non‐adjusted OR (95% CI)	Model I OR (95% CI)	Model II OR (95% CI)
		*p*	*p*	*p*
Low CRF (10.58–16.66)	143/236	1.00 (Reference)	1.00 (Reference)	1.00 (Reference)
Moderate CRF (16.67–20.90)	119/236	0.66 (0.46, 0.95) 0.026	0.42 (0.27, 0.64) < 0.001	0.56 (0.33, 0.95) 0.033
High CRF (20.92–34.93)	95/236	0.44 (0.30, 0.63) < 0.001	0.19 (0.12, 0.31) < 0.001	0.30 (0.17, 0.56) < 0.001
*p* for trend		< 0.001	< 0.001	< 0.001

*Note:* Non‐adjusted model adjusts for: None. Model I adjusted for age, sex. Model II adjusted for age, sex, body mass index, fasting plasma glucose, triglycerides, smoking status, drinking status, hypertension, dyslipidemia, diabetes, antiplatelet agents, antilipidemic agents, nitrates, calcium channel blockers, beta‐blockers, angiotensin converting enzyme inhibitors, angiotensin receptor blockers, angiotensin receptor neprilysin inhibitors, and hypoglycemic agents.

Abbreviations: CHD, coronary heart disease; CI, confidence interval; CRF, cardiorespiratory fitness; OR, odds ratio.

**FIGURE 1:**
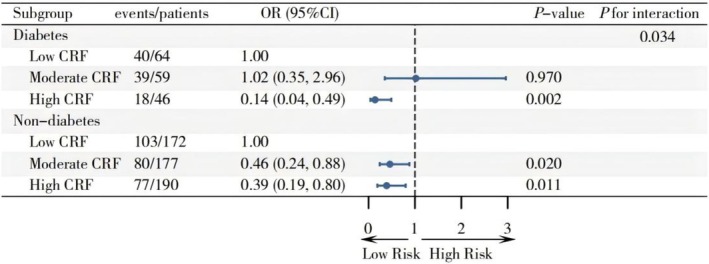
Effect modification of diabetes status on the association between CRF and CHD. Results were adjusted for age, sex, body mass index, fasting plasma glucose, triglycerides, smoking status, drinking status, hypertension, dyslipidemia, antiplatelet agents, antilipidemic agents, nitrates, calcium channel blockers, beta‐blockers, angiotensin converting enzyme inhibitors, angiotensin receptor blockers, angiotensin receptor neprilysin inhibitors, and hypoglycemic agents.

After stratification by diabetes status, a significant interaction was observed between CRF and diabetes status (*p* for interaction = 0.034). Among individuals with diabetes, only high CRF levels were associated with a significant reduction in CHD risk (OR = 0.14, 95% CI: 0.04–0.49; *p* = 0.002), while moderate CRF levels showed no significant effect (OR = 1.02, 95% CI: 0.35–2.96; *p* = 0.970). Conversely, in non‐diabetic individuals, moderate and high CRF levels were associated with significant reductions in CHD risk (OR = 0.46, 95% CI: 0.24–0.88; *p* = 0.020 and OR = 0.39, 95% CI: 0.19–0.80; *p* = 0.011), respectively, compared to low CRF levels. High CRF levels had a more pronounced impact on reducing CHD risk in diabetic patients compared to non‐diabetic patients (Figure 1).

## 3 Comment

This cross‐sectional study found an inverse association between CRF and CHD risk. Our result aligns with previous studies, further emphasizing the critical role of CRF in CHD prevention [4, 5]. To our knowledge, this is the first study to demonstrate that the association between CRF and CHD risk differs between non‐diabetic and diabetic individuals. Notably, the protective effect of high CRF levels on CHD risk is more pronounced in those with diabetes. Previous studies supported our findings [11, 12].

The following mechanisms may partly explain this effect. Diabetes, typically characterized by hyperglycemia and insulin resistance, induces oxidative stress, endothelial dysfunction, chronic inflammation, and dyslipidemia, which collectively significantly increase CHD risk [13, 14]. While moderate CRF may be insufficient to address this heightened pathological burden, high CRF can markedly improve insulin sensitivity, endothelial function, systemic inflammation, and body composition—directly targeting diabetes' core pathophysiology [15, 16, 17]. Notably, high‐CRF individuals often maintain healthier lifestyles, which further attenuate CHD risk in this population. These findings highlight the importance of improving CRF for CHD prevention, particularly among individuals with diabetes.

In our study, we used standardized objective measures, including CPET and CAG, to assess CRF and coronary stenosis severity. However, our findings can only establish associations rather than causality. Although we adjusted for multiple potential confounders, residual and unmeasured confounding may remain. Additionally, the generalizability of our findings to other populations may be limited. Future longitudinal studies with larger sample sizes, particularly in the diabetic subgroup, are needed to confirm the causal nature of these associations and to more robustly evaluate potential effect modification by diabetes status.

## Author Contributions

Li Wang designed the study, collected and analyzed the data, and wrote the manuscript. Yan Lu conceived the study, critically reviewed the manuscript, and contributed to editing. Huifang Zhang contributed to the conceptualization and methodology of the study.

## Funding

This work was supported by Shanxi Provincial Science and Technology Department (201903D321180, 202103021224393).

## Conflicts of Interest

The authors declare no conflicts of interest.

## Supporting information


**Table S1:** Demographic and clinical characteristics of participants with and without diabetes.

## Data Availability

The data cannot be made publicly available due to strict patient confidentiality restrictions.
