# Supplementary material for: Diabetes Status Modifies the Association Between Cardiorespiratory Fitness and Coronary Heart Disease: A Cross‐Sectional Study
Source: J Diabetes. 2025 Dec 5;17(12):e70176. doi: 10.1111/1753-0407.70176 (PMC12680446; doi:10.1111/1753-0407.70176)
Supplement: Supplementary file 1 — Table S1: Demographic and clinical characteristics of participants with and without diabetes. [file JDB-17-e70176-s001.docx]

**Table S1** Demographic and clinical characteristics of participants with and without diabetes.

| Variables | Total  (n = 708) | Without diabetes  (n = 539) | With diabetes  (n = 169) | *P*-value |
| --- | --- | --- | --- | --- |
| **Demographics** | | | | |
| Age (years) | 56.97 (9.55) | 56.32 (9.49) | 59.04 (9.48) | 0.001 |
| Sex |  |  |  |  |
| Male | 434 (61.30%) | 324 (60.11%) | 110 (65.09%) | 0.246 |
| Female | 274 (38.70%) | 215 (39.89%) | 59 (34.91%) |  |
| **Physical measurements** | | | | |
| BMI ( kg/m2) | 25.69 (3.04) | 25.48 (2.97) | 26.35 (3.17) | 0.001 |
| SBP (mmHg) | 119.95 (17.83) | 119.56 (17.05) | 121.20 (20.10) | 0.298 |
| DBP (mmHg) | 77.32 (11.39) | 77.60 (10.96) | 76.43 (12.64) | 0.244 |
| HR (bpm) | 75.12 (11.24) | 74.90 (11.27) | 75.81 (11.16) | 0.363 |
| **Behavioural** | | | | |
| Smoking |  |  |  |  |
| No | 493 (69.63%) | 373 (69.20%) | 120 (71.01%) | 0.656 |
| Yes | 215 (30.37%) | 166 (30.80%) | 49 (28.99%) |  |
| Drinking |  |  |  |  |
| No | 490 (69.21%) | 384 (71.24%) | 106 (62.72%) | 0.036 |
| Yes | 218 (30.79%) | 155 (28.76%) | 63 (37.28%) |  |
| **Biochemical measurements** | | | | |
| FPG (mmol/L) | 5.59 (1.68) | 5.01 (0.71) | 7.42 (2.43) | <0.001 |
| TC (mmol/L) | 4.21 (1.06) | 4.19 (1.02) | 4.28 (1.16) | 0.349 |
| TG (mmol/L) | 1.52 (1.10-2.14) | 1.47 (1.08-2.02) | 1.71 (1.24-2.44) | <0.001 |
| HDL-C (mmol/L) | 1.07 (0.25) | 1.09 (0.25) | 1.02 (0.23) | 0.003 |
| LDL-C (mmol/L) | 2.66 (0.77) | 2.64 (0.75) | 2.71 (0.83) | 0.319 |
| Creatinine (µmol/L) | 68.20 (13.25) | 68.56 (13.07) | 67.05 (13.80) | 0.197 |
| eGFR (mL/min/1.73m2) | 99.94 (11.18) | 100.03 (11.15) | 99.62 (11.31) | 0.678 |
| **Comorbidities** | | | | |
| Hypertension |  |  |  |  |
| No | 315 (44.49%) | 272 (50.46%) | 43 (25.44%) | <0.001 |
| Yes | 393 (55.51%) | 267 (49.54%) | 126 (74.56%) |  |
| Dyslipidemia |  |  |  |  |
| No | 463 (65.40%) | 364 (67.53%) | 99 (58.58%) | 0.033 |
| Yes | 245 (34.60%) | 175 (32.47%) | 70 (41.42%) |  |
| **Cardiorespiratory fitness** | | | | |
| VO_2_peak (mL/min/kg) | 19.13 (4.49) | 19.40 (4.66) | 18.28 (3.80) | 0.005 |
| **CHD** | | | | |
| No | 351 (49.58%) | 279 (51.76%) | 72 (42.60%) | 0.038 |
| Yes | 357 (50.42%) | 260 (48.24%) | 97 (57.40%) |  |
| **Medication use at discharge** | | | | |
| Antiplatelet |  |  |  |  |
| No | 153 (21.61%) | 135 (25.05%) | 18 (10.65%) | <0.001 |
| Yes | 555 (78.39%) | 404 (74.95%) | 151 (89.35%) |  |
| Antilipidemic |  |  |  |  |
| No | 94 (13.28%) | 90 (16.70%) | 4 (2.37%) | <0.001 |
| Yes | 614 (86.72%) | 449 (83.30%) | 165 (97.63%) |  |
| Nitrates |  |  |  |  |
| No | 501 (70.76%) | 393 (72.91%) | 108 (63.91%) | 0.025 |
| Yes | 207 (29.24%) | 146 (27.09%) | 61 (36.09%) |  |
| CCB |  |  |  |  |
| No | 492 (69.49%) | 389 (72.17%) | 103 (60.95%) | 0.006 |
| Yes | 216 (30.51%) | 150 (27.83%) | 66 (39.05%) |  |
| Beta-blockers |  |  |  |  |
| No | 368 (51.98%) | 297 (55.10%) | 71 (42.01%) | 0.003 |
| Yes | 340 (48.02%) | 242 (44.90%) | 98 (57.99%) |  |
| ACEI/ARB/ANRI |  |  |  |  |
| No | 423 (59.75%) | 347 (64.38%) | 76 (44.97%) | <0.001 |
| Yes | 285 (40.25%) | 192 (35.62%) | 93 (55.03%) |  |
| Hypoglycemic |  |  |  |  |
| No | 559 (78.95%) | 530 (98.33%) | 29 (17.16%) | <0.001 |
| Yes | 149 (21.05%) | 9 (1.67%) | 140 (82.84%) |  |

Note: Values were expressed as mean(SD), median (Q1-Q3), or number (percentage).

Abbreviations: BMI, body mass index; SBP, systolic blood pressure; DBP, diastolic blood pressure; HR, heart rate; FPG, fasting plasma glucose; TC, total cholesterol; TG, triglycerides; HDL-C, high-density lipoprotein cholesterol; LDL-C, low-density lipoprotein cholesterol; eGFR, estimated glomerular filtration rate; VO_2_peak, Peak Oxygen Uptake; CHD, coronary heart disease; CCB, calcium channel blockers; ACEI, angiotensin converting enzyme inhibitors; ARB, angiotensin receptor blockers; ARNI, angiotensin receptor neprilysin inhibitors.
